# Improving Electrochromic Cycle Life of Prussian Blue by Acid Addition to the Electrolyte

**DOI:** 10.3390/ma12010028

**Published:** 2018-12-21

**Authors:** ZiTong Li, YunHui Tang, KaiLing Zhou, Hao Wang, Hui Yan

**Affiliations:** The College of Materials Science and Engineering, Beijing University of Technology, Beijing 100124, China; xukun188@emails.bjut.edu.cn (Z.L.); zkling@emails.bjut.edu.cn (K.Z.); hyan@bjut.edu.cn (H.Y.)

**Keywords:** electrochromism, Prussian blue, cycle life

## Abstract

In this study, we examined the cyclic stability of Prussian blue (PB) films in electrolytes with acid. The cyclic stabilities of the PB films were investigated in K^+^ based electrolytes with different values of solution pH. The acidified KCl solution can significantly improve the durability of the film. Among the three pH values tested, the KCl solutions (pH = 2.15 and pH = 3.03) showed better performance. Furthermore, we investigated the cyclic stabilities of the PB films in LiClO_4_/PC electrolyte containing different acids. We found that the cyclic stability of PB film was significantly improved when a small amount of acetic acid was dissolved in LiClO_4_/PC electrolyte. The PB film exhibited stable optical modulation after up to 20,000 cycles in LiClO_4_/PC electrolyte containing acetic acid—a much higher result than those of some literatures. This suggests that the addition of acetic acid to LiClO_4_/PC electrolyte can promote the development of PB-based devices with improved stability.

## 1. Introduction

Electrochromism refers to the phenomenon that materials can switch their optical properties reversibly by applying an external potential or current [1]. Recently, numerous electrochromic materials have been investigated for their high optical modulation, great stability, and simple preparation, such as WO_3_, NiO, and polyaniline [2,3,4,5,6,7]. These materials have been widely applied for various applications such as switchable windows [8], electrochromic displays [9], rechargeable batteries [10], and optical attenuators [11].

Fe complexes [12,13,14,15] also exhibit potential applications in the field of electrochromism. Among these Fe complexes, Prussian blue (PB, an iron (II,III) hexacyanoferrate (II,III)) gains much attention for its high redox reversibility and low threshold potential [16]. Prussian blue is a coordination compound, which can change optical properties by intercalating or de-intercalating ions. Many electrochromic devices (ECD) employ PB as the complementary electrochromic material. Examples include PB/tungsten oxide [17,18], PB/poly (butyl viologen) [19], and PB/poly (3,4-ethylenedioxythiophene) (PEDOT) [20]. However, the cycle life of the PB film is generally only about 100 cycles. The poor durability of Prussian blue films restricts its applications in an electrochromic field. Some researchers tried to solve this problem by improving the preparation method. Qian et al. [21] reported that nanostructured PB films could be grown using a template-free hydrothermal technique. The film possessed cycling stability after 150 cycles. Wang et al. [22] synthesized PB film via an electrochemical post-treatment procedure. The resulting PB film exhibited improved stability in neutral and alkaline solutions after 500 cycles. Meanwhile, some researchers tried to synthesize a PB hybrid film. Cheng et al. [23] fabricated a nanocomposite Prussian blue (NPB) film composed of the random packing of ITO nanoparticles. The NPB film exhibited a high optical modulation and slight charge density decay after 2000 cycles. Seelandt et al. [24] used a TiO_2_ thin film possessing an ordered array of mesopores to support PB. The PB/TiO_2_ hybrid film had high optical modulation (ΔT = 65%) and fast switching speed (T_c_ = 3 s, T_b_ = 2 s) after 400 cycles. In addition, some researchers tried to improve the electrolyte performance. Wang et al. [18] reported the synthesis of a poly (methyl methacrylate)-succinonitrile composite polymer electrolyte which was then applied to WO_3_/PB complementary electrochromic devices. The ΔT of this device lost 15% of its original value after 2250 cycles. Nonetheless, the methods mentioned above do not significantly improve the cycle life of the PB film, and the film generally exhibits obvious optical modulation decay after 2500 cycles.

In this work, the cyclic stabilities of PB films were investigated in K^+^-based electrolytes with different values of solution pH. Meanwhile, we investigated the influence of adding different acids (hydrochloric acid and acetic acid) to LiClO_4_/PC electrolyte on the cycle life of the PB films. The cyclic stability of the PB films exhibited significant improvement when acetic acid was dissolved in LiClO_4_/PC electrolyte.

## 2. Experimental

### 2.1. Materials

All the chemicals were used without further purification. Potassium ferricyanide, acetone, ethanol, hydrochloric acid, acetic acid, iron (III) chloride, and potassium chloride were all obtained from Beijing Chemical Works. Lithium perchlorate and propylene carbonate were purchased from Damao Chemical Reagent Factory. Indium tin oxide (ITO) glasses (3 × 4 cm^2^ in size and sheet resistant Rs ≈ 20 Ω) were used as the substrates. All experiments were performed at room temperature in air.

### 2.2. Sample Preparation

PB films were deposited on the ITO substrates that were cleaned in acetone, ethanol, and deionized water successively using ultrasound. In a typical route, a plating solution of PB film was prepared, consisting of 10 mM K_3_[Fe(CN)_6_], 10 mM FeCl_3_, and 0.1 M KCl with dilute HCl to adjust to the pH to 2. ITO substrates, a Pt plate, and an Ag/AgCl electrode were used as the working electrode, the counter electrode, and the reference electrode, respectively. The electroplating was carried out galvanostatically for 15 min by applying cathodic current densities of 10 µA/cm^2^ at room temperature. After the deposition, the samples were washed by distilled water and then dried in air before proceeding with future experiments.

### 2.3. Characterization and Electrochemical Measurements

The morphologies of the films were measured by scanning electron microscope (SEM, FEI Quanta 650). The cyclic voltammetry (CV) and chronoamperometry measurements were carried out using a Princeton VersaSTAT 4 electrochemical workstation in a three-electrode electrochemical cell with Ag/AgCl as the reference electrode, PB as the work electrode, Pt plate as the counter electrode, and 1 M KCl solution or 1 M LiClO_4_/propylene carbonate (PC) as the electrolyte. The in situ observation of the optical transmittance of the PB films at 690 nm was measured using an ultraviolet-visible-near-infrared spectrophotometer (Shimadzu UV-3101PC) combined with a square quartz groove (5.5 × 5.5 × 5 cm^3^) during all electrochemical cycling.

## 3. Results and Discussion

**Figure 1:**
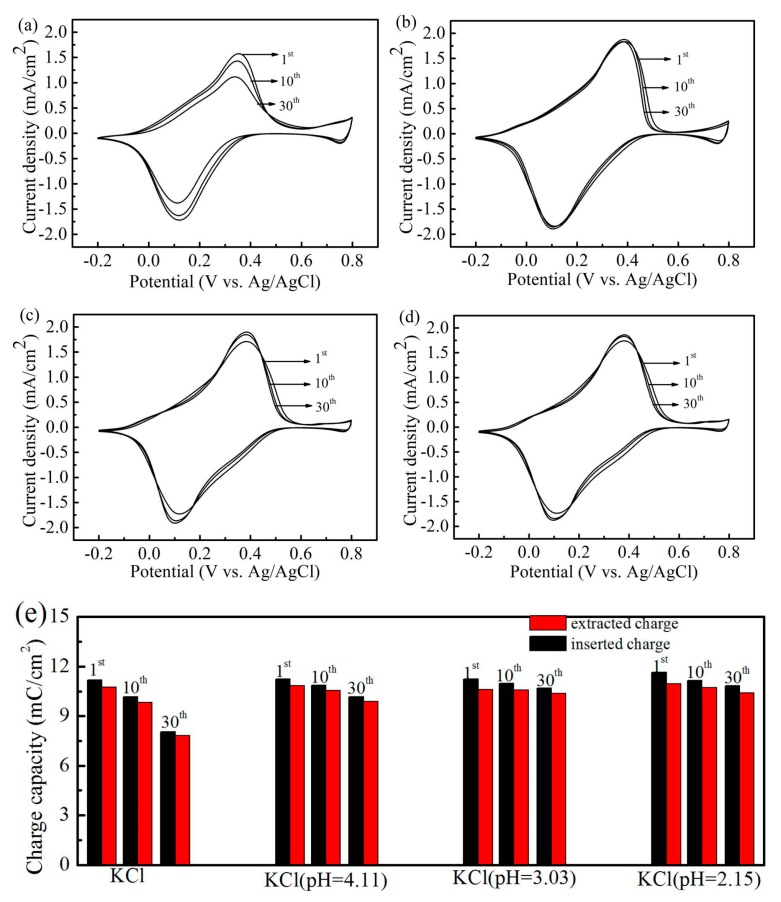
Cyclic voltammograms of Prussian blue (PB) film in (**a**) neutral 1 M KCl solution, and KCl aqueous solution with different values of solution pH: (**b**) 4.11, (**c**) 3.03, and (**d**) 2.15. (**e**) Inserted and extracted charge densities versus cycle number derived from the cyclic voltammetry (CV) data.

Figure 1a–d show the CV curves of the PB film in neutral and acidified KCl solution. The applied potential is set between −0.2 and 0.8 V relative to the Ag/AgCl reference electrode with the potential sweep rate of 50 mV/s. As shown in Figure 1a, there are two redox peaks: one at 0.14 V (PB/Prussian white (PW)) and another at 0.8 V (PB/Prussian yellow (PY)). The two redox peaks are identified as PB reducing to PW and as PB oxidizing to PY, as in the following reactions [25]:

(1) $$KFe^{⫼}[Fe^{\|}{\left(CN\right)}_{6}]+K^{+}+e^{-}↔K_{2}Fe^{\|}[Fe^{\|}{\left(CN\right)}_{6}]$$

PB PW

(2) $$KFe^{⫼}[Fe^{\|}{\left(CN\right)}_{6}]↔Fe^{⫼}[Fe^{⫼}{\left(CN\right)}_{6}]+K^{+}+e^{-}$$

PB PY

The PB film shows definite separations of voltammetric peaks for the reduction and oxidation, which can be attributed to the resistivity of the ITO substrate layer [26]. In addition, the peak current density of PB/PW is higher than that of PY/PB in the testing. On the one hand, the PB structure only contains 3Fe^2+^ sites (involved in PB/PY) by each 4Fe^3+^ (involved in PB/PW), so not all oxidized Fe^2+^ may be balanced by cations [27]. On the other hand, the CV curves were measured in the potential cycled from −0.2 to 0.8 V. The redox reaction of PB/PY is not sufficient in this range.

As shown in Figure 1a, we failed to establish any long cycling stability of the PB film in neutral KC1 solutions due to the strong interaction between ferric ions and hydroxide ions. However, the remarkable durability of the film was observed in acidified KCl solutions, and these results are consistent with those reported in some research works [28]. Figure 1b–d show the CV curves of the PB films in acidified KCl solution with hydrochloric acid; the values of the solution pH are 4.11, 3.03, and 2.15, respectively. The PB films retained their CV shape without significant decay in 30 cycles compared to Figure 1a,e, which show the charge capacity, calculated by integrating the insertion/extraction parts of CV data, during 30 cycles. Our results show a slight difference between inserted and extracted charges in the one cycle. This indicates that the partially inserted ions cannot completely be extracted in one cycle. We attribute this phenomenon to the insufficiency of the PB/PY reaction. The charge capacity of the 30th cycle was 72% of the initial value in neutral KCl solution. The values tested in acidified KCl solution exceeded 90% and were clearly higher than those of the neutral KCl solution. The results imply that the acidified KCl solution can significantly improve the durability of the film, and the KCl solutions (pH = 2.15 and pH = 3.03) show better performance.

**Figure 2:**
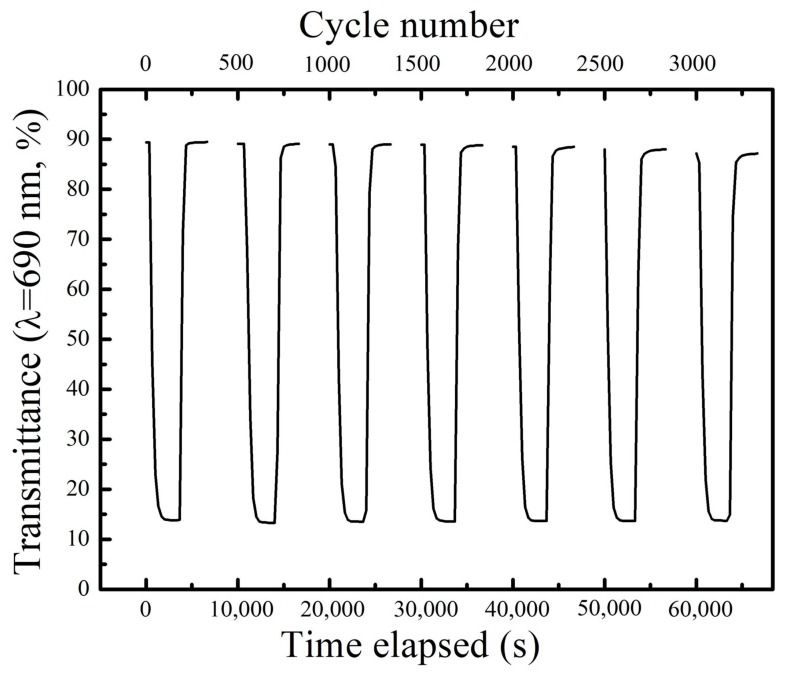
Evolution of optical transmittance spectra at 690 nm for Prussian blue (PB) film under a step potential varied from −0.05 V to 0.5 V for 10 s in KCl (pH = 3.03) solution.

The potential steps of −0.05 and 0.5 V were applied for 10 s to investigate the cyclic stability of the PB film in KCl (pH = 3.03). The corresponding in situ transmittance responses at 690 nm are shown in Figure 2. There was no significant degradation of the PB film after 3000 cycles.

When the acidified KCl solution was employed as an electrolyte for the PB-based ECD, it was difficult to seal the ECD completely to prevent solution leakage. Furthermore, we cannot neglect the security issue of this ECD because the value of the KCl solution pH is 3. So, replacing the acidified KCl solution with LiClO_4_/PC electrolyte is a promising option. In the following part, the cyclic stability of the PB film in LiClO_4_/PC electrolyte is investigated. Meanwhile, we discuss the influence of adding strong acid and weak acid to LiClO_4_/PC electrolyte on the cyclic stability of the PB film. The volume ratio of LiClO_4_/PC to acetic acid was 200:1; the volume ratio of LiClO_4_/PC to hydrochloric acid was the same as above.

**Figure 3:**
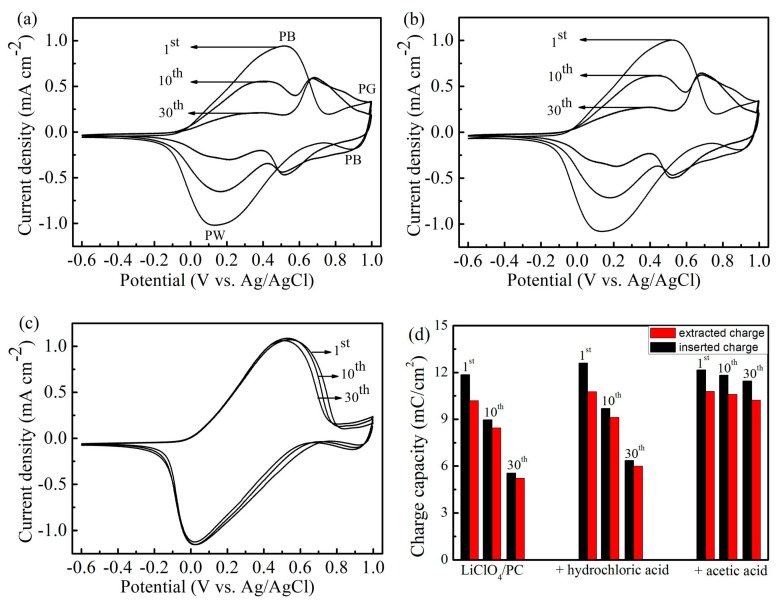
The cyclic voltammetry (CV) evolutions of the Prussian blue (PB) film in LiClO_4_/PC electrolytes with different acid: (**a**) LiClO_4_/PC, (**b**) LiClO_4_/PC/HCl, and (**c**) LiClO_4_/PC/HAc. (**d**) Inserted and extracted charge densities versus cycle number derived from CV data.

Figure 3 shows the CV curves of the PB film in LiClO_4_/PC electrolytes with different levels of acid. Compared with Figure 1, the shift of anodic peaks and cathodic peaks shown in Figure 3 indicates that the redox reaction of the film is diffusion-limited [29]. The interfacial reaction kinetics and transport rate all influence the cycle life of the film. The resistivity of Li^+^ transfer through the interface between the solid electrode and the polymer electrolyte is different from the resistivity of K^+^ transfer through solid/liquid interfaces [30]. The ionic radius of K^+^ and Li^+^ is different, so the rate of ion insertion/extraction is different. Figure 3a shows the CV results of the PB film tested in pure LiClO_4_/PC electrolyte. The peak current density of PB/PW becomes lower while the peak current density of PB/PY becomes higher in the cycling. In addition, the area of CV curves decreases as the number of cycles increases. The results reveal that the redox reaction of PB/PW gradually weakens after the 30th cycle. Figure 3b shows the CV curves of the film tested in LiClO_4_/PC electrolyte containing hydrochloric acid (LiClO_4_/PC/HCl). The results are similar to the former and indicate that the addition of hydrochloric acid has no significant effect on the stability of the PB film. Figure 3c shows the results of the PB film tested in LiClO_4_/PC electrolyte containing acetic acid (LiClO_4_/PC/HAc). The areas of CV curves and redox peaks of PB film exhibit no obvious difference between the first and the 30th cycle. The evolution of the charge density of the films is shown in Figure 3d; it can be clearly seen that the charge density of the film cycling in LiClO_4/_PC/HAc remains at 94.2% of the original value, which is much higher than the two other experimental results. The results imply that the addition of acetic acid can improve the durability of the PB film in LiClO_4_/PC electrolyte.

Large optical modulation and long-term durability are the key prerequisites for the practical implementation of electrochromic devices [31]. Here, we employed chronoamperometry and in situ optical transmittance measurements to estimate the influence of different acids (hydrochloric acid and acetic acid) on the cycle life of the PB film in LiClO_4_/PC electrolyte.

**Figure 4:**
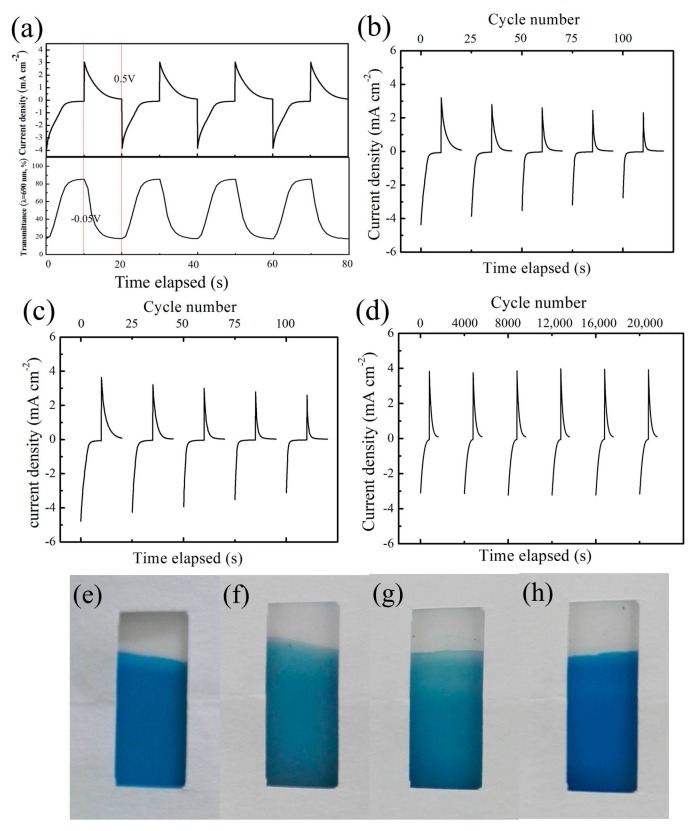
(**a**) In situ observation of the transmittance at λ = 690 nm at the potential steps of −0.05 V and 0.5 V. Chronoamperometric curve evolution of the Prussian blue (PB) film in (**b**) LiClO_4_/PC, (**c**) LiClO_4_/PC/HCl, and (**d**) LiClO_4_/PC/HAc. (**e**) The photograph of the as-electrodeposited PB film. The photographs of the PB film after (**f**) 100 cycles in LiClO_4_/PC, (**g**) 100 cycles in LiClO_4_/PC/HCl, and (**h**) 20,000 cycles in LiClO_4_/PC/HAc.

As shown in Figure 4a, the in situ optical transmittance at 690 nm and chronoamperometric responses of the PB films on cycling were synchronously recorded. An upward transmittance curve and negative current responses were recorded when the negative potential was applied; the reduction of Fe^Ⅲ^ to Fe^Ⅱ^ led to the bleaching of the PB film. A downward transmittance curve and positive current responses were recorded when the positive potential was applied; the oxidation of Fe^Ⅱ^ to Fe^Ⅲ^ led to the coloration of the film. Figure 4b shows the chronoamperometric curves of the PB film in LiClO_4_/PC. The peak current densities of the oxidation and reduction reaction reached a maximum at the first cycle and then decreased gradually upon cycling. This indicates that the PB film gradually loses its electrochromic activity in the cycling. Figure 4c shows the curves of the PB film tested in LiClO_4_/PC/HCl. The results of the chronoamperometric evolution are similar to Figure 4b, which illustrates that hydrochloric acid cannot improve the durability of PB film in LiClO_4_/PC electrolyte. Figure 4d shows the results of the PB film tested in LiClO_4_/PC/HAc. The peak current densities exhibited no obvious changes upon switching. The cycle life of the PB film tested in Li^+^-based electrolyte exceed 20,000 cycles, which is much higher than the results reported in some research works. The results indicate that the PB film tested in LiClO_4_/PC/HAc possesses stable electrochromic activity. Figure 4e–h show the photographs of the as-electrodeposited PB film and the film after testing in LiClO_4_/PC electrolyte containing different acids. Distinct color fading of the film was observed after 100 cycles in LiClO_4_/PC electrolyte and LiClO_4_/PC/HCl. However, the PB film after 20,000 cycles in LiClO_4_/PC/HAc showed no obvious color change compared to the as-deposited PB film. These results imply that suitable acidic environments can inhibit the decomposition of PB film in LiClO_4_/PC electrolyte and are consistent with the data from the chronoamperometric tests.

**Figure 5:**
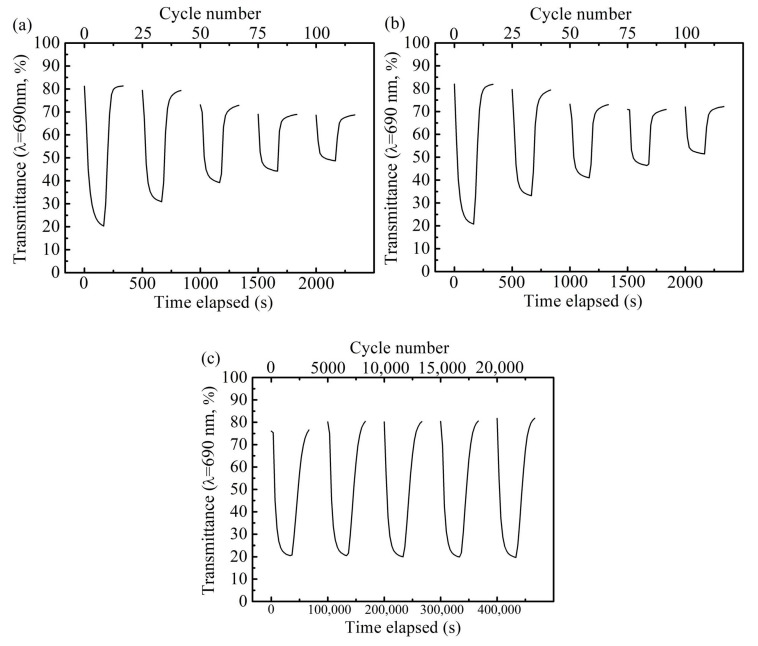
Evolution of the optical transmittance spectra at 690 nm for the Prussian blue (PB) film in (**a**) LiClO_4_/PC, (**b**) LiClO_4_/PC/HCl, and (**c**) LiClO_4_/PC/HAc.

Figure 5a shows the evolution of optical transmittance spectra at λ = 690 nm of the PB film cycling in LiClO_4_/PC electrolyte. The initial optical transmittance of the PB film in the bleaching and coloration states was 83.2% and 22.3%, respectively; hence, the optical modulation was 60.9%. However, both the bleaching and coloration curves decayed rapidly with the cycling. The optical modulation was only 20.0% at the 100th cycle, which is 32.8% of the initial cycle. Figure 5b shows the results of the PB film tested in LiClO_4_/PC/HCl. The decay tendency of the optical modulation is similar to that in Figure 5a. In detail, the decreased speed of coloration state is slightly lower in the first 25 cycles than that in Figure 5a. Figure 5c shows the evolution of optical transmittance spectra of the PB film cycling in LiClO_4_/PC/HAc. There was no obvious degradation of the electrochromic properties after 20,000 cycles. The optical modulation of the PB film at the 20,000th cycle was 60.8%, which is slightly higher than the 57.5% of the first cycle.

**Figure 6:**
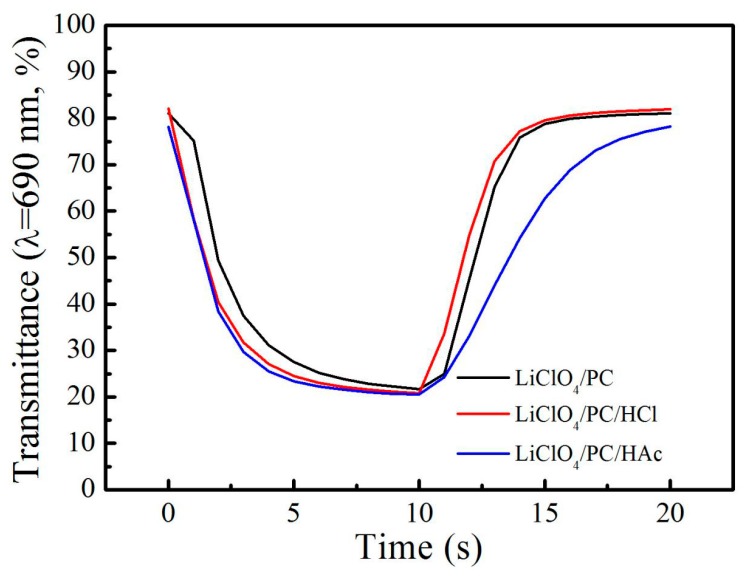
The transmittance response at 690 nm with switching potential between −0.05 V and 0.5 V for 10 s.

The switching speed at 690 nm measured from the time dependence of transmittance responses is shown in Figure 6. The switching speed time is defined as the 90% interval between the bleaching state and coloration state. The coloration time of the film in the three electrolytes is almost the same (3.5 s), while the bleaching time in LiClO_4_/PC/HAc is 7.1 s, which is obviously larger than the 4 s of the other two cases. These results indicate that the addition of acetic acid can greatly improve the cycle life of the PB film, but it reduces the bleaching speed of the film.

**Figure 7:**
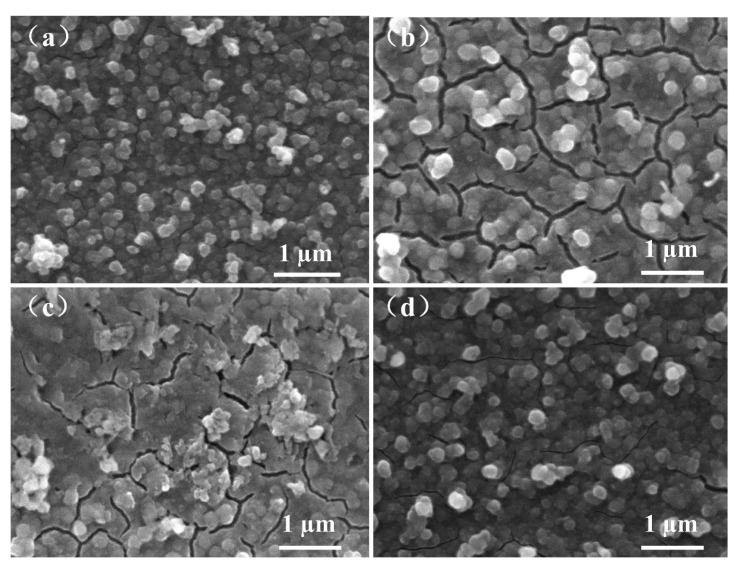
(**a**) The SEM image of the as electrodeposited Prussian blue (PB) film. SEM images of the PB film after (**b**) 100 cycles in LiClO_4_/PC, (**c**) 100 cycles in LiClO_4_/PC/HCl, and (**d**) 20,000 cycles in LiClO_4_/PC/HAc.

Figure 7a shows the SEM image of the as-electrodeposited PB film. Some particle-like clusters were observed, which can be attributed to the formation of agglomerates during the film deposition. Besides, the film had some cracks between particle-like clusters. Figure 7b shows the SEM image of the film after 100 cycles in LiClO_4_/PC electrolyte. The cracks of the film became wider and longer. The SEM result of the PB film after 100 cycles in LiClO_4_/PC/HCl is shown in Figure 7c, which is similar to the feature of Figure 7b. Figure 7d shows the surface morphology of the PB film after 20,000 cycles in LiClO_4_/PC/HAc. There is no significant difference in the surface morphology of the film after testing compared with that of the as-deposited PB film. The results indicate that the structure of the film was stable during the Li^+^ insertion and extraction processes. The suitable acidic environment can inhibit the destruction of the PB film upon cycling.

The reversibility of the film is expressed as the ratio of the extracted charges (Q_ex_) to inserted charges (Q_in_) during the bleaching and coloration processes as follows [32]:

(3) $$\mathrm{Reversibility}=\frac{Q_{ex}}{Q_{in}}\times 100\%$$

**Figure 8:**
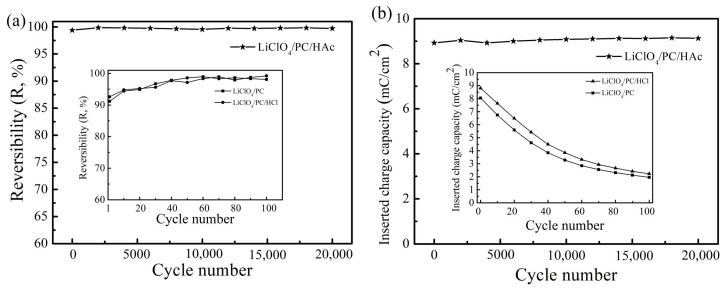
(**a**) Evolution of the reversibility; (**b**) the inserted charge capacity of the PB film in different electrolytes.

Figure 8a shows the evolution of the reversibility of the PB film in the three electrolytes. The films cycling in the three electrolytes all exhibited impressive reversibility (more than 91%), and a slight increase of the reversibility was observed in the cycling. The results imply that most of the inserted charges can be extracted in the following oxidation step. Figure 8b shows the evolution of inserted charge capacities of the PB films in the three electrolytes. The inserted charge capacity of the PB film tested in LiClO_4_/PC electrolyte was about 1.9 mC/cm^2^ at 100th cycle, which is only 23.6% of the initial cycle. This implies that the charge capacity of the film was greatly reduced after 100 cycles, which led to the decrease of the electrochromic activity of the film. The PB film, KFe[Fe(CN)_6_], generally contains an indeterminate amount of water in the interstices of the cubic lattice structure [33]. Meanwhile, the LiClO_4_/PC electrolyte contains a small amount of water molecules. This suggests that the strong interaction between ferric ions and hydroxide ions forms Fe(OH)_3_, resulting in the destruction of the Fe-CN-Fe bond and in the solubilization of the PB film [34]. The reaction can be written as follows [35]:

From the above results we can see that a cycle lifetime in excess of 20,000 was achieved in LiClO_4_/PC electrolyte containing acetic acid and was much higher than the results of some previously reported works. This suggests that a suitable acidic environment can inhibit the reaction between Fe^3+^ and OH^−^ to form Fe(OH)_3_, and that the structure of the film is stable during the Li^+^ insertion and extraction processes. However, the inserted charge capacity of the PB film tested in LiClO_4_/PC/HCl obviously decreased as the cycle number increased, indicating that the hydrochloric acid cannot prevent the decomposition of PB film in LiClO_4_/PC electrolyte. This suggests that H^+^ ions can inhibit the reaction between Fe^3+^ and OH^-^ to form Fe(OH)_3_, but excess H^+^ ions in hydrochloric acid will participate in the redox reaction of the PB film together with Li^+^ ions, which can destroy the lattice structure of the film [36]. The reaction can be written as follows:

(5) $$XFe^{⫼}[Fe^{\|}{\left(CN\right)}_{6}]+X^{+}+e^{-}↔X_{2}Fe^{\|}[Fe^{\|}{\left(CN\right)}_{6}]$$

where *X* represents Li^+^ and H^+^ ions.

## 4. Conclusions

In summary, the electrochromic performances of the PB films in K^+^-based electrolytes with different solution pH values were investigated. Acidified KCl solutions can significantly improve the durability of the PB film, and the KCl solutions (pH = 2.15 and pH = 3.03) showed better performance. Furthermore, we investigated the cycle stabilities of PB films in LiClO_4_/PC electrolyte containing different acids. The cyclic stability of the PB film did not significantly improve when the hydrochloric acid was dissolved in LiClO_4_/PC electrolyte. Hydrochloric acid partly prevents the film from decomposing during the cycling. However, it may be involved in the redox reaction of PB and could destroy the lattice structure of the film. The film cycling in LiClO_4_/PC with acetic acid electrolyte exhibited no obvious transmittance fade after 20,000 cycles and had a much greater cycle life than some previously demonstrated PB films. This suggested that the method of adding acetic acid to the electrolyte provided an effective way to improve the cyclic stability of the PB film in LiClO_4_/PC.
